# Prevention programs

**DOI:** 10.1007/s00068-025-02832-8

**Published:** 2025-03-26

**Authors:** Luca Fattori, Roman Pfeifer

**Affiliations:** 1 Department of General and Emergency Surgery, School of Medicine and Surgery, University of Milano-Bicocca, Monza, Italy; 2 Department for Traumatology, University Hospital Zurich, Zurich, Switzerland

**Keywords:** Whitebook, Polytrauma, ESTES

## Abstract

With a rise in traumatic incidents across Europe, there is an escalating need for a proactive and coordinated approach to trauma prevention. This chapter outlines evidence-informed strategies and collaborative efforts aimed at reducing the incidence and impact of trauma in Europe.

## Introduction

Effective trauma prevention begins with a thorough understanding of its causes and consequences. Trauma results from a variety of mechanisms, including road traffic injuries (RTIs), falls, violence, and natural disasters. A robust public health approach must identify and address risk factors, promote safer environments, and advocate for policies that prioritise injury prevention.

## The role of healthcare professionals

Healthcare professionals play a central role in trauma prevention. Beyond emergency response and rehabilitation, they encourage preventive measures such as promoting healthier lifestyles, regular check-ups, and safety awareness. Improving the quality and accessibility of trauma care, particularly in underserved areas, can significantly reduce the impact of traumatic events.

Integrating **Traumatic Injury Prevention Programs (TIPP)** into healthcare systems reduces injuries and deaths across all populations. Fostering trauma-informed care within the healthcare sector also addresses the long-term consequences of trauma, including psychological repercussions.

## The role of lead agencies

Lead agencies coordinate prevention strategies at regional, state, and local levels. By collaborating with public health authorities, community organisations, scientific societies, and the private sector, they can develop, implement, and evaluate injury prevention programs. Evidence-informed strategies based on systematic epidemiologic data ensure targeted and effective interventions.

Collaborative partnerships across sectors enhance efficiency and impact. Establishing an **injury control network**—a broad alliance involving healthcare, professional, and community organisations—facilitates coordinated efforts. Specific attention must be given to vulnerable populations, including children, older adults, and others at heightened risk.

### Levels of prevention

Trauma prevention efforts operate at three distinct levels:

- **Primary prevention**: Aimed at the entire population to decrease the overall risk of injury (e.g., civil engineering guidelines, window guards, smoke detectors).
- **Secondary prevention**: Targeted at high-risk populations to mitigate the effects of traumatic events (e.g., car seats, seat belts, helmets).
- **Tertiary prevention**: Focused on reducing the long-term impact of trauma on individuals and communities (e.g., EMS and trauma systems support, rehabilitation, access to care).

Prevention strategies must be tailored to the needs of local communities, with adequate funding, staffing, and partnerships to ensure successful implementation.

## Conclusion and needs for the future

Preventing trauma in Europe requires a proactive, evidence-based approach integrating public health initiatives, education, and healthcare services. Key priorities include:

- Embedding prevention strategies into public policies.
- Focusing on education, training, and safety promotion.
- Enhancing healthcare services, particularly in underserved areas.
- Addressing root causes of trauma through collaboration across sectors.

By fostering a comprehensive and coordinated strategy, Europe can achieve a significant reduction in trauma incidence, safeguarding public health and improving the quality of life for individuals across all communities.

## Data Availability

No datasets were generated or analysed during the current study.
